# Encapsulation of *Verbascum sinaiticum* Leaf Extract as a Natural Antimicrobial for Controlling Microbial Growth in Beef During Refrigerated Storage

**DOI:** 10.3390/molecules31122063

**Published:** 2026-06-12

**Authors:** Alemu Belay Legesse, Shimelis Admassu Emire, Timilehin Martins Oyinloye, Won Byong Yoon

**Affiliations:** 1 School of Chemical and Bioengineering, Addis Ababa Institute of Technology, Addis Ababa University, P.O. Box 385, King George VI Street, Addis Ababa 16417, Ethiopia; alemubelay@dbu.edu.et (A.B.L.); shimelis.admassu@aait.edu.et (S.A.E.); 2 Department of Food Engineering, College of Engineering, Debre Berhan University, Debre Berhan P.O. Box 445, Ethiopia; 3 Department of Food Science and Biotechnology, College of Agriculture and Life Sciences, Kangwon National University, Chuncheon 24341, Republic of Korea; oyinloyetm@kangwon.ac.kr; 4 Elder-Friendly Research Center, Agriculture and Life Science Research Institute, Kangwon National University, Chuncheon 24341, Republic of Korea; 5 Department of Food Biotechnology and Environmental Science, Kangwon National University, Chuncheon 24341, Republic of Korea

**Keywords:** *V. sinaiticum*, natural antimicrobials, encapsulation, maltodextrin, gum arabic, beef preservation, food safety, refrigerated storage

## Abstract

The efficacy of plant-derived antimicrobials in meat systems is frequently limited by interactions with proteins, lipids, and other food matrix components that reduce the bioavailability and antimicrobial activity of phytochemicals. This study evaluated the antimicrobial effectiveness of *Verbascum sinaiticum* (*V. sinaiticum*) leaf extract encapsulated using maltodextrin (MD), gum arabic (GA), and a maltodextrin–gum arabic blend (MDGA, 8:2 *w*/*w*) through freeze-drying for application in raw beef during refrigerated storage (4 °C). The encapsulation systems exhibited process yields of 42.5–54.7%, encapsulation efficiencies of 78.3–92.5%, and loading capacities of 18.5–24.3 mg GAE/g DW, with MDGA showing the highest encapsulation efficiency. The effects of encapsulation on microbial inhibition, physicochemical properties, and sensory quality were investigated over 15 days of storage. Aerobic plate counts in the control increased from 3.04 to 8.26 log CFU/g, whereas encapsulated treatments showed significantly lower final counts (*p* < 0.05), reaching 7.89 log CFU/g (MD), 7.96 log CFU/g (MDGA), and 7.95 log CFU/g (GA). Similarly, encapsulated treatments reduced *Escherichia coli* counts during storage, with maltodextrin (MD) exhibiting the greatest inhibitory effect (6.23 × 10^5^ CFU/g) compared with the control (6.93 × 10^5^ CFU/g) on day 15. However, reductions in *Staphylococcus aureus*, *E. coli*, *Candida albicans*, and *Bacillus cereus* remained below 1 log CFU/g, indicating limited antimicrobial efficacy under the tested conditions. All encapsulated treatments slowed pH increases during storage (6.20–6.34) relative to the control (6.62) on day 15 and preserved aroma quality throughout the storage period. Overall, encapsulation improved the antimicrobial performance of *V. sinaiticum* extract compared with the free extract, particularly in MD-based systems; however, the antimicrobial effects in beef remained modest. These findings highlight both the potential and current limitations of encapsulated plant-derived antimicrobials for meat preservation and emphasize the need for optimized delivery systems to enhance efficacy in complex food matrices.

## 1. Introduction

Growing consumer demand for natural and sustainable preservatives is driving a transformation in the food industry, shifting the focus from synthetic additives to plant-derived alternatives. While synthetic preservatives effectively control spoilage and pathogenic microorganisms, increasing concerns regarding their potential health risks and environmental impact have intensified the pursuit of safer, natural alternatives [1,2]. This trend is particularly relevant in meat preservation, where natural antimicrobials align with consumer preferences for clean-label products while enhancing shelf life and safety.

Among plant-based antimicrobial agents, *V. sinaiticum* has garnered attention due to its traditional medicinal use and documented analgesic and antimicrobial properties [3,4]. The plant is a perennial medicinal herb widely distributed in East Africa, particularly in Ethiopia. In Ethiopian traditional medicine, different parts of the plant, especially the leaves and roots, have been used for the treatment of wounds, respiratory disorders, gastrointestinal ailments, skin infections, inflammation, and livestock diseases [4,5,6]. Rich in bioactive compounds such as saponins, phenolic acids, and terpenoids, *V. sinaiticum* has been reported to disrupt microbial membranes and metabolic processes, positioning it as a potential antimicrobial agent [5]. Phytochemical investigations have identified compounds including verbascoside (acteoside), catechin derivatives, kaempferol derivatives, myricetin derivatives, and proanthocyanidins, which contribute to its biological activity [4,5,6]. These phytochemicals may exert antimicrobial effects through disruption of microbial cell membranes, interference with enzyme activity, induction of oxidative stress, and inhibition of essential metabolic pathways. Recent studies have demonstrated its activity against a broad spectrum of microorganisms, including *Bacillus cereus*, *Staphylococcus aureus*, *Escherichia coli*, and *Candida albicans* [4,6,7], supporting its potential application as a natural antimicrobial in food preservation.

However, the direct application of plant leaf extracts in food systems is often limited by instability, sensitivity to environmental conditions, and low bioavailability [8,9]. Encapsulation provides a viable solution by entrapping bioactive compounds within protective matrices, enhancing stability, enabling controlled release, and improving overall efficacy in food preservation [9,10,11]. Techniques such as freeze-drying, spray drying, coacervation, extrusion, and electrospinning are commonly employed to protect sensitive ingredients and improve solubility, sensory attributes, and stability in food products [12].

Among coating materials, maltodextrin (MD) and gum arabic (GA)—utilized individually or in synergy—stand as the gold standard in microencapsulation due to their complementary physicochemical and functional profiles. MD is valued for its high aqueous solubility, low viscosity, and capacity to form robust amorphous matrices, which facilitates the thermal protection of labile bioactive compounds [9,13]. Conversely, GA offers superior emulsifying capacity and interfacial stabilization, which is critical for reducing the oxidative degradation of sensitive extracts. The combination of coating materials has been widely investigated for its ability to improve encapsulation efficiency, enhance powder stability, and facilitate the controlled release of bioactive compounds during storage and application. Furthermore, freeze-drying was prioritized over conventional thermal methods for this microbiological application. By utilizing low-temperature sublimation, freeze-drying prevents the thermal denaturation of thermolabile antimicrobial metabolites within the *V. sinaiticum* extract, thereby preserving the maximum inhibitory potential of the encapsulated powder against foodborne pathogens in the beef matrix.

Among the various encapsulation drying techniques, spray drying and freeze-drying are the most commonly used because of their efficiency in preserving and protecting bioactive compounds. Freeze-drying is widely recognized as a preferred method because of its superior capacity to preserve heat-sensitive and oxidation-prone bioactive compounds, thereby maximizing antimicrobial activity and enhancing product stability during processing and storage. Unlike conventional thermal drying methods such as spray drying, freeze-drying operates under low-temperature and low-pressure conditions, thereby minimizing thermal degradation, volatilization losses, Maillard reactions, and structural collapse of the encapsulating matrix. This preservation of chemical integrity is particularly critical in food microbiology applications, where the retention of antimicrobial efficacy and functional stability of bioactive compounds directly influences microbial inhibitory performance [9,13]. Consequently, freeze-drying ensures higher retention of bioactivity and improved functional performance of the encapsulated system for food preservation and microbial control applications.

The total phenolic content (TPC) of plant extracts is widely recognized as a key indicator of their antimicrobial and antioxidant potential, as phenolic compounds such as tannins and flavonoids can scavenge free radicals and inhibit microbial growth [6]. Encapsulation has been reported to enhance the stability of TPC during storage and processing, thereby preserving the bioactivity of plant-derived compounds [14,15]. In meat systems, encapsulated plant extracts can improve thermal stability and provide sustained antimicrobial activity against foodborne pathogens, contributing to extended shelf life and improved product safety [1,2,15,16,17,18,19]. This work provides new insights into the potential of encapsulated plant extracts as natural preservatives in meat products.

Therefore, the primary objective of this study was to evaluate the antimicrobial efficacy of encapsulated *V. sinaiticum* leaf extract against key foodborne microorganisms, including *Bacillus cereus*, *Escherichia coli*, *Candida albicans*, and *Staphylococcus aureus* in beef. A secondary objective was to characterize the physicochemical properties of the encapsulated extract, including thermal stability, color, aroma, and textural attributes, using MD, GA, and their combination (MDGA) as carrier materials. This study aims to contribute to the development of plant-based antimicrobial systems for meat preservation, supporting improved food safety, shelf-life extension, and sustainable processing strategies.

## 2. Results and Discussion

### 2.1. Extraction and Encapsulation: Preparation and Characterization

The maceration extraction of *V. sinaiticum* using 70% ethanol resulted in an extraction yield of 25.85 ± 0.08% and a total phenolic content (TPC) of 181.50 ± 0.12 mg GAE/g dw (Table 1). The relatively high TPC indicates that aqueous ethanol is an effective solvent system for recovering phenolic compounds, likely due to its optimal polarity for solubilizing both hydrophilic and moderately lipophilic phytochemicals. These findings are in agreement with previous reports demonstrating the efficiency of hydroalcoholic solvents in extracting phenolics from medicinal plants [9].

Encapsulation performance was evaluated in terms of process yield (PY), encapsulation efficiency (EE), and loading capacity (LC). The PY ranged from 42.5 ± 1.2% to 54.7 ± 1.3%, with significantly higher values observed for GA and the combined MDGA system (*p* < 0.05). The improved PY in GA-containing formulations can be attributed to its superior film-forming ability and reduced stickiness during drying, which enhances powder recovery. The highest PY obtained for MDGA (54.7%) suggests a synergistic interaction between MD and GA, leading to improved encapsulation matrix formation.

EE, which reflects the retention of phenolic compounds within the encapsulating matrix, was consistently high across all treatments, ranging from 78.3 ± 2.5% to 92.5 ± 1.7%. The MDGA formulation exhibited the highest EE (92.5%), indicating enhanced protection of phenolic compounds. This synergistic effect may be explained by the complementary functional properties of MD and GA: MD contributes to film formation and solubility, while GA improves emulsification and stabilizes bioactive compounds during processing. The combination, therefore, minimizes phenolic degradation and surface exposure [7,8,9,13].

Similarly, LC followed an increasing trend, with MDGA showing the highest value of 24.3 ± 0.9 mg GAE/g dw, although no statistically significant difference (*p* > 0.05) was observed among encapsulant types within this parameter. The higher LC in MDGA further confirms its superior ability to entrap and retain phenolic compounds within the matrix, enhancing the functional value of the encapsulated powder.

Overall, the results demonstrate that both GA and MD are effective encapsulating agents; however, their combination (MDGA) provides superior performance in terms of PY, EE, and LC. This highlights the importance of selecting appropriate wall material combinations to optimize encapsulation efficiency and bioactive compound retention. The present findings are consistent with previous studies, which reported PY values ranging from 32.55% to 55.00% and EE values between 68.67% and 97.13% for plant extract encapsulation systems [20].

### 2.2. Physicochemical Characterization

#### 2.2.1. Thermal Properties

**Figure 1:**
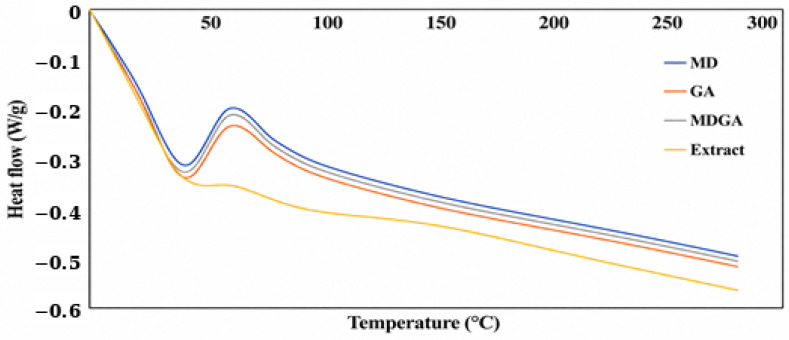
Thermograms of the pure wall materials and obtained encapsulates: MD—maltodextrin encapsulates, GA—gum arabic encapsulates, and a combination of maltodextrin and gum arabic (8:2 *w*/*w*) (blend) encapsulates, as well as the non-encapsulated, freeze-dried extract (Extract).

The thermal behavior of *V. sinaiticum* leaf extract and its encapsulated forms (MD, GA, and their combination (MDGA)) was evaluated using differential scanning calorimetry (DSC) (Figure 1). All samples exhibited an initial endothermic event within the temperature range of approximately 50–150 °C, which is commonly attributed to the evaporation of residual and loosely bound moisture. The non-encapsulated extract displayed a broader and more intense endothermic peak compared to the encapsulated samples, suggesting a higher moisture content and a greater proportion of unbound or weakly bound water. In contrast, the encapsulated formulations (MD, GA, and MDGA) showed relatively reduced peak intensity and slight shifts in peak temperature. This behavior may indicate interactions between the extract and wall materials, which can influence water mobility and retention within the matrix. Such effects have been widely reported in polysaccharide-based encapsulation systems, where hydrogen bonding and matrix structuring contribute to modified thermal responses [21].

Following the initial moisture loss event, the DSC thermograms of the encapsulated samples (MD, GA, and MDGA) exhibited subtle baseline deviations that may be indicative of glass-transition-related phenomena. However, since the glass transition temperatures (Tg) were neither directly measured nor quantitatively determined, interpretation of these thermal features must be approached with caution. Consequently, no definitive conclusions can be drawn regarding Tg behavior or the comparative thermal stability of the investigated systems.

The observed baseline deviations may be attributed to the presence of amorphous domains within the encapsulating matrices, which is consistent with the established physicochemical properties of carbohydrate-based wall materials such as MD and GA. Furthermore, residual moisture within the system can act as a plasticizing agent, influencing thermal behavior by lowering the apparent Tg and broadening the transition range. Such moisture-induced plasticization effects are well documented in carbohydrate-rich systems and are known to significantly affect thermal transitions [22].

Nevertheless, in the absence of explicit Tg determination—such as midpoint Tg values obtained from conventional DSC or more sensitive techniques like modulated DSC—these interpretations should be considered tentative. Therefore, assertions regarding smoother thermal transitions or enhanced thermal stability among the encapsulated samples should be avoided or expressed with appropriate caution unless supported by quantitative thermal data.

The enthalpy changes in the DSC curves reflect the energy required for the phase transitions or degradation processes. The free extract exhibited the highest overall enthalpy changes, indicating significant energy absorption with temperature. Conversely, encapsulation with MD and GA mitigated this issue by forming a protective matrix around the bioactive compounds, thereby delaying the onset of thermal degradation. The MDGA combination demonstrated superior thermal resistance, as evidenced by the sustained heat flow at higher temperatures and potentially shifted degradation events with in the temperature range. This suggests synergistic stabilization effects from the blended (MDGA) encapsulants.

The DSC results underscore the critical role of encapsulation in stabilizing heat sensitive bioactive compounds within *V. sinaiticum* extract. The improved moisture binding, altered glass transition behavior, and enhanced thermal resistance observed in the encapsulated forms (GA, MD, and MDGA), particularly with the MDGA combination, confirm its effectiveness as a protective formulation for maintaining the integrity and efficacy of the extract’s functional properties and providing superior thermal protection.

#### 2.2.2. Antimicrobial Activity

The antimicrobial activities of GA, MD, MDGA encapsulated formulations, and *V. sinaiticum* leaf extract were evaluated against *Staphylococcus aureus* (SA), *Escherichia coli* (EC), *Candida albicans* (CA), and *Bacillus cereus* (BC) using the agar disc diffusion method. Antimicrobial efficacy was assessed by measuring the inhibition zone diameter (IZD, mm) at concentrations of 0.5, 1.0, 1.5, and 2.0 mg/mL. Ciprofloxacin was used as a positive control to benchmark antimicrobial performance.

**Figure 2:**
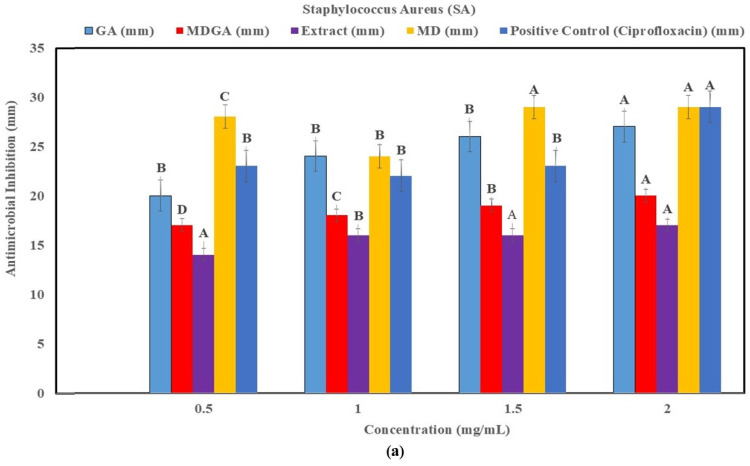

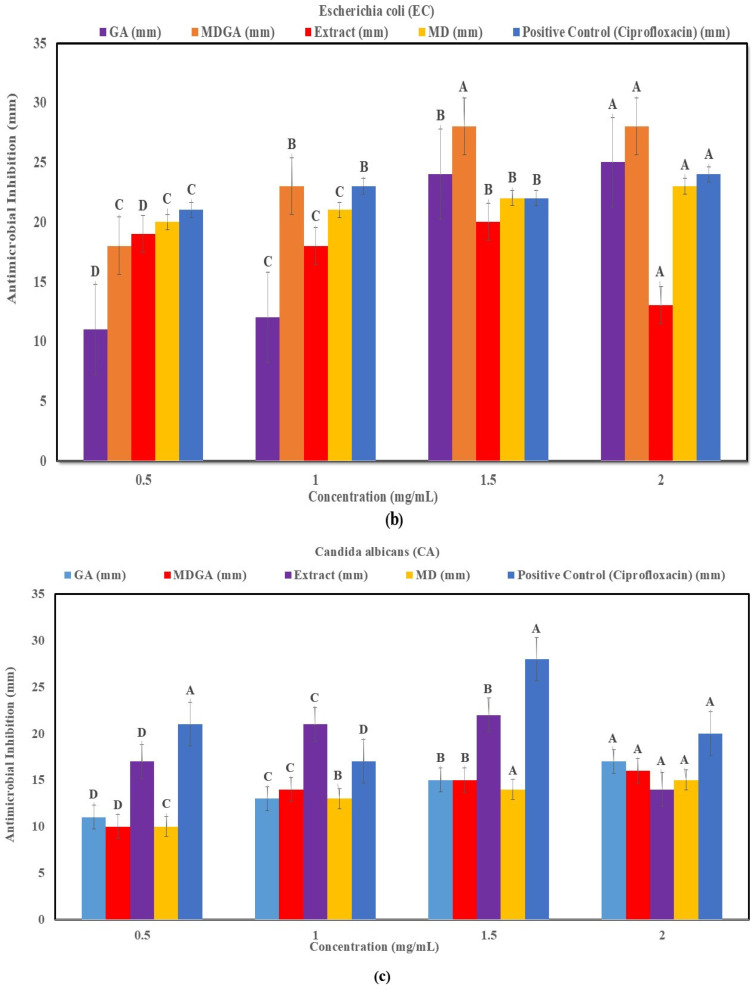

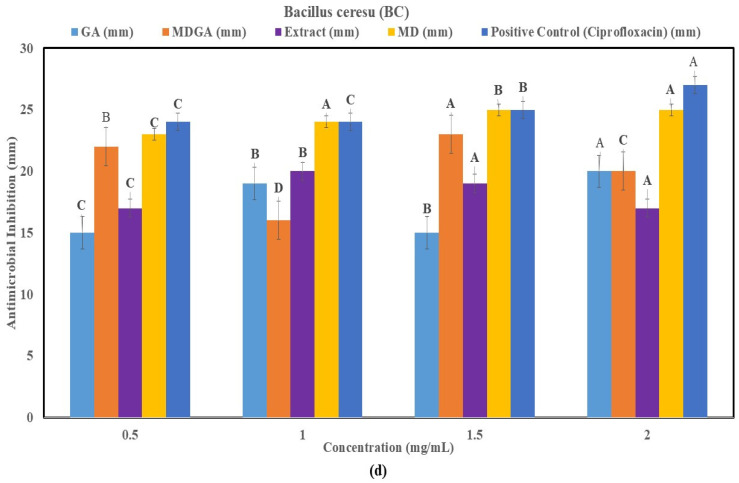
Antimicrobial inhibition of GA, MD, MDGA extract, and ciproflaxin (positive control): (**a**) *Stephylococcus aurus*; (**b**) *E. Coli*; (**c**) *Candida albicans*; (**d**) *Bacillus cereus*. Different uppercase letter from the bars indicate significant differences among treatments at the same concentration (*p* < 0.05), while bars with the same letter are not significantly different.

**Figure 3:**
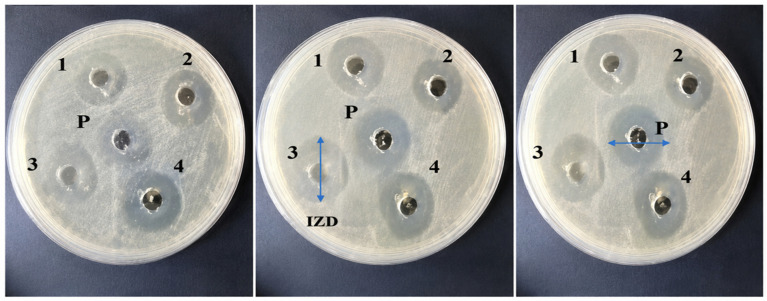
Antimicrobial inhibition of *V. sinaiticum* leaf extract and encapsulated Inhibition Zone Diameters (mm) for different microbial strains. 1:0.5 mg/mL, 2:1 mg/mL, 3:1.5 mg/mL, and 4:2 mg/mL, P: Positive control (ciprofloxacin), and IZD: Inhibition Zone Diameters, 1–4: antimicrobial inhibition, ⟷: inhibition zone diameter.

The quantitative results are presented in Figure 2, while representative agar plate images illustrating inhibition zones and measurement procedures are shown in Figure 3. It is important to note that inhibition zone diameters provide a diffusion-based estimate of antimicrobial activity and should not be interpreted as Minimum Inhibitory Concentration (MIC) values, which require agar dilution methods under standardized conditions.

As shown in Figure 2, all treatments exhibited a clear concentration-dependent increase in inhibition zone diameter, indicating enhanced antimicrobial activity with increasing dosage. This trend was consistently observed across all tested microorganisms, although the degree of susceptibility varied among species. Gram-positive bacteria (*S. aureus* and *B. cereus*) generally showed greater sensitivity compared to Gram-negative *E. coli*, which may be attributed to the protective outer membrane structure that limits the diffusion of antimicrobial compounds [4,6]. *Candida albicans* exhibited moderate susceptibility, reflecting differences in cell wall composition and permeability.

Among the tested treatments, MD and MDGA formulations demonstrated comparatively larger inhibition zones, suggesting enhanced antimicrobial efficacy. This effect can be attributed to the role of encapsulation in improving the stability, protection, and controlled release of bioactive compounds present in *V. sinaiticum* extract. The synergistic effect of MD and GA as wall materials likely contributed to improved dispersion and sustained release of active compounds, thereby increasing their interaction with microbial cells.

Figure 3 further supports these findings, showing well-defined inhibition zones surrounding the discs and confirming the antimicrobial activity of the tested formulations. The clear visualization of inhibition zones and the consistency between visual and quantitative data reinforce the reliability of the measurements.

This indicates that encapsulation enhances the antimicrobial performance of *V. sinaiticum* extract, with MDGA formulations showing particular promise for application in food preservation systems. However, it should be emphasized that inhibition zone diameters reflect diffusion-dependent antimicrobial effects and provide only a semi-quantitative assessment. This trend is similarly to the previous study [10,23].

The results demonstrated a clear concentration-dependent antimicrobial activity for all tested formulations, as indicated by the progressive increase in inhibition zone diameter (IZD) with increasing concentration. Among the encapsulating agents, MD consistently exhibited the highest antimicrobial efficacy across the tested microbial strains. Against Staphylococcus aureus (Figure 2a), MD produced the largest inhibition zones, reaching 29.14 mm at 2.0 mg/mL, compared with GA (27.28 mm) and the free extract (22.18 mm). Similar trends were observed for the other tested microorganisms, although the extent of inhibition varied depending on microbial type and susceptibility.

The comparatively larger inhibition zones observed for MD-based formulations may be associated with improved stabilization and dispersion of bioactive compounds within the encapsulating matrix. Polysaccharide-based carriers such as MD have been reported to enhance the apparent bioactivity of plant-derived compounds by protecting sensitive constituents and maintaining their functional integrity during processing and application [8,24]. In this context, the improved performance of MD formulations in the present study likely reflects enhanced preservation and availability of active compounds during the assay.

However, it is important to emphasize that the agar disc diffusion method provides a diffusion-dependent estimate of antimicrobial activity and does not allow direct inference of specific mechanisms of action. Therefore, the observed differences in inhibition zone diameter should not be interpreted as definitive evidence of enhanced cellular penetration, membrane disruption, or controlled release behavior. Instead, these results reflect the combined influence of antimicrobial potency and the physicochemical properties of the encapsulating materials, including their solubility and diffusion characteristics in the agar medium.

Furthermore, the variability in antimicrobial response among the tested microorganisms may be attributed to differences in cell wall structure and permeability. For Gram-positive bacteria such as *S. aureus* are generally more susceptible to plant-derived antimicrobials than Gram-negative bacteria, which possess an outer membrane that can limit compound diffusion.

Overall, the findings suggest that encapsulation, particularly using MD, enhances the apparent antimicrobial performance of *V. sinaiticum* extract in a disc diffusion system. However, further investigation using standardized minimum inhibitory concentration (MIC) assays and mechanistic studies is necessary to confirm these observations and to better understand the mode of action of the encapsulated bioactive compounds [4,23].

For *E. coli* (Figure 2b), MDGA showed exceptional activity (28.31 mm at 1.5 mg/mL), possibly due to improved diffusion, stabilization, or availability of active compounds [16]. The extract’s moderate efficacy (19–21 mm) suggests partial susceptibility of Gram-negative bacteria to unencapsulated bioactive.

Against *C. albicans* (Figure 2c), MD again outperformed other formulations (28.12 mm at 1.5 mg/mL), while GA and MDGA had limited efficacy (≤17 mm). This mirrors findings by [25], where MD’s controlled release improved antifungal action.

*B. cereus* (Figure 2d) was most inhibited by MD (25.18 mm at 2 mg/mL), with MDGA also showing promise (23.16 mm at 1.5 mg/mL). The extract’s lower activity (≤20 mm) underscores encapsulation’s role in enhancing bioavailability [4].

Encapsulation improved the apparent antimicrobial activity of *V. sinaiticum* extract in the agar disc diffusion assay, with MD showing the largest inhibition zones among the tested carriers. While these findings indicate enhanced performance under in vitro screening conditions, they should be interpreted as diffusion-dependent effects rather than direct evidence of antimicrobial efficacy in real food systems.

The observed improvement may be associated with better stabilization and availability of bioactive constituents within the encapsulated matrices. Previous studies indicate that *V. sinaiticum* contains a range of phenolic and flavonoid compounds, including catechin, umbelliferone, kaempferol, and myricetin derivatives; verbascoside; and proanthocyanidins [4,6,26]. In the present study, however, the absence of compound-specific quantification precludes attribution of the observed antimicrobial activity to individual constituents. The activity is therefore more appropriately interpreted as arising from the combined effects of these bioactive compounds, with phenolic rich fractions—particularly verbascoside and proanthocyanidins—likely contributing substantially.

Overall, the findings indicate that encapsulation significantly enhanced the in vitro antimicrobial activity of *V. sinaiticum* extract compared with the non-encapsulated extract (*p* < 0.05). Among the encapsulating agents, MD-based formulations exhibited superior antimicrobial performance, which may be attributed to improved protection and controlled release of bioactive compounds. However, further studies involving compositional characterization, standardized minimum inhibitory concentration (MIC) and minimum bactericidal concentration (MBC) determinations, and validation in food systems are necessary to confirm efficacy and elucidate the underlying mechanisms before practical application as a natural food preservative.

### 2.3. Effect of V. sinaiticum Extract Treatments on Beef Quality During Storage

#### 2.3.1. Texture Properties

Table 2 summarizes the textural properties of beef samples treated with *V. sinaiticum* leaf extract (free) and encapsulated forms (GA, MD, and MDGA) during storage at 4 °C (days 1, 3, and 9). The evaluated parameters included adhesiveness (N), gumminess (N), and chewiness (N), as well as springiness and cohesiveness (dimensionless). Changes in these parameters are commonly associated with microbial spoilage processes, particularly the activity of slime-producing bacteria and fungi, which can alter meat structure by increasing surface stickiness (adhesiveness) and reducing elasticity (springiness) and internal bonding (cohesiveness) [27]. In the present study, samples treated with encapsulated *V. sinaiticum* extracts showed a slower rate of textural deterioration compared to untreated controls and those treated with free extract.

Among the formulations, the MDGA combination exhibited the most consistent preservation of textural attributes over the 9-day storage period, suggesting improved stability under both refrigeration and ambient conditions. This effect may be associated with the protective role of encapsulation matrices, which can enhance the retention and availability of bioactive compounds during storage. Similar observations have been reported for plant extract encapsulation in biopolymers such as MD and GA, where improved preservation of food quality parameters has been linked to reduced microbial activity [28]. Therefore, while the observed improvements in texture are consistent with reduced spoilage-related degradation, a direct causal relationship between microbial inhibition and textural preservation cannot be conclusively established without complementary microbiological analyses.

Springiness, which reflects the elastic recovery of the sample after deformation, remained relatively stable across all treatments throughout storage, with values ranging from approximately 0.004 to 0.023 (Table 2). This indicates that there was no substantial loss of elastic structural integrity during storage in either encapsulated or non-encapsulated samples. The stability of springiness suggests that the coating systems may have contributed to maintaining the protein–polysaccharide matrix by limiting structural collapse and delaying protein denaturation, in agreement with previous studies by [29,30]. Importantly, springiness is a dimensionless parameter, and any previous reporting in force units is incorrect and has been corrected accordingly. Cohesiveness reflects the internal structural integrity of meat and its ability to withstand deformation during compression. Encapsulated samples, particularly the MDGA treatment, showed relatively higher cohesiveness values at later storage stages compared with initial measurements. For example, cohesiveness increased from 0.0064 on Day 1 to 0.0169 on Day 9, suggesting improved maintenance of structural integrity during refrigerated storage.

In contrast, samples treated with the free extract exhibited a gradual increase in cohesiveness over time (reaching 0.055 by Day 9), indicating a more moderate and progressive effect on structural stabilization. These changes may be associated with differences in moisture retention and matrix interactions within the meat system; however, such interpretations should be made cautiously, as cohesiveness is influenced by multiple factors, including protein denaturation, water distribution, and storage conditions [31]. Overall, the results suggest that encapsulation, particularly with MDGA, may contribute to improved textural stability during storage.

Gumminess and chewiness increased significantly across all treatments during refrigerated storage (Table 2), with the most pronounced changes observed in MDGA- and extract-coated samples. For instance, gumminess values in MDGA-treated samples increased markedly from Day 1 to Day 9, while extract-treated samples exhibited the highest overall values at later storage stages.

These increases in gumminess and chewiness indicate progressive structural changes in the meat matrix during storage. Such changes may be associated with moisture re-distribution, protein denaturation, and alterations in muscle fiber integrity. The presence of coating materials, particularly polysaccharide-based systems such as MD and GA, may influence these properties by forming surface barriers that modify water mobility and mass transfer.

However, the observed increase in firmness-related parameters should be interpreted with caution. While moderate increases in gumminess and chewiness may contribute to perceived textural stability, excessive increases can indicate undesirable toughening or partial dehydration of the meat matrix rather than a purely beneficial preservation effect. Therefore, the higher values observed in MDGA- and extract-treated samples at extended storage times may reflect a combination of protective coating effects and moisture loss dynamics.

Previous studies have reported that polysaccharide-based coatings can influence textural attributes by altering water retention and structural integrity [32,33]. In agreement with these findings, the present results suggest that encapsulation systems can modulate texture during storage. Nevertheless, the extent of hardening observed, particularly at later storage stages, highlights the need to balance preservation benefits with potential impacts on consumer acceptability.

Coating treatments affect the evolution of textural properties during storage; however, increases in gumminess and chewiness should not be interpreted solely as indicators of improved preservation. Instead, they reflect complex interactions between moisture retention, structural changes, and storage conditions, which require careful optimization to maintain desirable meat quality.

#### 2.3.2. Color Analysis

Color was evaluated using the CIE L*a*b* color system, where L* indicates lightness, a* redness, b* yellowness, and ΔE represents the total color difference relative to the initial storage day. Measurements were conducted over a 15-day refrigerated storage period for beef samples treated with different formulations (Control, MD, MDGA, and GA) (Table 3). As shown in Table 3, the lightness (L*) of all samples decreased significantly (*p* < 0.05) during refrigerated storage, indicating progressive darkening of the meat. The control sample exhibited the most pronounced decline, decreasing from 35.09 on day 1 to 26.04 on day 15. In contrast, all treated samples (MD, MDGA, GA, and extract) retained higher L* values throughout storage, suggesting improved preservation of lightness. Among the treatments, MD (28.89) and MDGA (28.72) showed slightly higher final L* values compared to GA (28.22) and the free extract (28.03), indicating a modest advantage in maintaining brightness.

The redness parameter (a*) declined significantly across all treatments during storage, reflecting the progressive degradation of oxymyoglobin and the associated loss of the desirable red color (Table 3). The control samples exhibited a pronounced decrease, from 21.3 on day 1 to 8.8 on day 15, indicating substantial color deterioration.

All treated samples (MD, MDGA, GA, and extract) showed a similar declining trend, although the rate of decrease was moderately reduced compared to the control. For example, GA-treated samples decreased from 18.93 to 7.17 over the storage period. However, the differences among treatments at later storage stages were relatively small, and in some cases, the final a* values of encapsulated treatments were comparable to or slightly lower than those of the control.

These findings suggest that while the treatments may have contributed to a modest delay in redness loss during early storage, they did not fully prevent pigment degradation over time. The observed trends may be associated with the presence of bioactive compounds in the extract, which have been reported to influence oxidative stability in meat systems [34,35].

The b* values in the control group decreased from 7.66 to 4.07, indicating a significant reduction in yellowness (Table 3). Conversely, the encapsulated treatments maintained higher b* values, particularly MD, which began at 8.12 and ended at 5.51. The higher *CIE b* values observed in the encapsulated treatments, particularly GA, indicate improved retention of yellowness during storage. This effect may be related to the ability of the encapsulation matrix to protect pigments and phenolic compounds from oxidative degradation. By limiting oxidation, encapsulation may help preserve color attributes and enhance the overall color stability of the meat samples during refrigerated storage [36,37].

The ∆E values reflect the overall color change during storage. The control sample showed a clear decrease in ∆E from 63.04 (day 1) to 38.85 (day 15), indicating progressive color change over time rather than an increase, as previously stated. This reduction suggests that the magnitude of color difference relative to the initial state diminished during storage, likely due to simultaneous changes in multiple color coordinates (L*, a*, and b*). In comparison, the encapsulated treatments also exhibited decreasing ∆E values over time, with GA declining from 62.46 (day 1) to 42.95 (day 15) (Table 3). Importantly, higher ∆E values at later storage stages indicate greater color deviation from the reference state, whereas lower ∆E values suggest relatively better color stability. In this context, the control sample reached the lowest ∆E value at day 15, while encapsulated treatments maintained comparatively higher values, indicating differences in color evolution patterns among treatments.

Therefore, the interpretation of ∆E must be made cautiously, as lower values do not necessarily indicate improved color preservation without considering the reference point and overall color trajectory. The encapsulated treatments (MD, MDGA, and GA) appeared to moderate the rate of color change across storage, as supported by their more gradual decline in ∆E values. The observed effects may be associated with the presence of bioactive compounds in *V. sinaiticum*, which have been reported to exhibit antioxidant properties that can influence pigment stability in meat systems. However, these effects should be interpreted conservatively, as ∆E represents a composite color parameter and does not directly quantify specific oxidative or pigment degradation mechanisms. The encapsulated extracts contributed to maintaining color attributes during storage, particularly by moderating changes in lightness, redness, and yellowness. This stabilization is important for preserving the visual quality and consumer acceptability of meat products during refrigerated storage by reducing oxidative reactions and extending shelf life.

#### 2.3.3. Evaluating Changes in Beef Quality During Storage at 4 °C

Statistical analysis revealed significant differences (*p* < 0.05) among treatments (control, MD, MDGA, GA, and extract) and storage time for pH, aerobic plate counts (APCs), *Escherichia coli*, and aroma (Table 4). These findings confirm that both treatment type and storage duration significantly influenced microbial dynamics and sensory quality of beef during refrigerated storage.

Microbiological criteria for fresh meat in the Republic of Korea specify limits of ≤1.0 × 10^7^ CFU/g for aerobic plate count (APC) and ≤1.0 × 10^3^ CFU/g for *Escherichia coli* (*E.coli*), as outlined by the Ministry of Food and Drug Safety (MFDS, 2023; guideline No. 2014-135). In comparison, the mean *E. coli* count reported for beef samples from meat packing centers in the Gangwon region was 6.26 × 10^5^ CFU/g, exceeding the recommended limit.

The pH of beef samples increased progressively during storage at 4 °C, which is consistent with microbial spoilage characterized by the accumulation of alkaline metabolites such as ammonia and biogenic amines [38,39]. The control sample exhibited a marked increase from 5.64 to 6.34 by day 15, exceeding the commonly accepted spoilage threshold (pH > 6.2) for fresh meat [40,41]. However, Table 4 presents only a single pH dataset, while the discussion implies treatment-specific differences. This discrepancy should be clarified. If pH was measured for all treatments, the complete dataset should be presented; otherwise, conclusions regarding comparative treatment effects on pH should be interpreted cautiously.

Despite this limitation, the observed trend suggests that treatments likely contributed to delaying pH increase, possibly through inhibition of microbial metabolism. Similar effects of plant-derived antimicrobials in retarding pH rise have been widely reported [7,16,42,43].

The initial APC values (2.94–3.04 Log CFU/g) indicate good microbiological quality of fresh beef. However, a significant increase (*p* < 0.05) was observed across all treatments during storage. The control reached 8.26 Log CFU/g by day 15, exceeding the widely accepted spoilage limit of 6–7 Log CFU/g and indicating advanced deterioration [35,43,44].

Treated samples (MD, MDGA, GA, and extract) showed slightly lower APC values (7.89–8.01 Log CFU/g at day 15), demonstrating a moderate antimicrobial effect. Among treatments, MD exhibited the lowest APC, suggesting that encapsulation may enhance antimicrobial efficacy through improved stability and controlled release of bioactive compounds [45]. Nevertheless, all treatments approached spoilage thresholds by the end of storage, indicating that the inhibitory effect was limited rather than absolute.

A similar trend was observed for *E. coli*, with counts increasing throughout storage. The control showed the highest levels (5.48 × 10^5^ to 6.93 × 10^5^ CFU/g), exceeding the microbiological limit of ≤1.0 × 10^3^ CFU/g recommended by [41]. Treated samples, particularly MD, exhibited comparatively lower counts, indicating partial inhibition of coliform growth. However, the relatively high initial contamination suggests either substantial baseline microbial load or an experimental challenge condition, which should be clearly stated to contextualize antimicrobial efficacy [46].

Microbial contamination of meat arises at multiple stages along the supply chain, including transportation, storage, and handling at retail outlets [16,35,47]. These factors highlight the importance of effective preservation and hygiene practices to ensure microbial safety.

Aroma scores declined significantly (*p* < 0.05) in all treatments during storage, reflecting progressive spoilage. Initial scores (~8.5) indicated excellent freshness, but values dropped below the acceptability threshold (<3) after day 12, consistent with microbial growth and accumulation of off-odor volatile compounds [48].

The control exhibited the most pronounced decline (8.50 to 1.60), whereas treated samples showed slightly slower deterioration (e.g., MD: 8.50 to 1.75). Although some treatments (MD, MDGA) demonstrated marginally higher aroma scores at early storage stages, differences were not consistently significant across all time points. Therefore, no definitive conclusion can be drawn regarding superior sensory preservation.

The combined microbiological and sensory results showed a clear relationship between microbial growth and quality deterioration during refrigerated storage. Aerobic plate counts (APCs) increased significantly (*p* < 0.05), reaching 6.89–7.01 Log CFU/g on day 12 and 7.89–8.26 Log CFU/g on day 15. Although 7 Log CFU/g is widely accepted as the microbiological spoilage limit for fresh meat, aroma acceptability declined significantly (*p* < 0.05) before this threshold was reached. Sensory deterioration became evident when APC values approached 6 Log CFU/g, suggesting that this level may better represent consumer acceptance under the conditions studied. Similar observations were reported by [35], who found sensory quality loss before microbial counts reached conventional spoilage limits. Likewise, [40] highlighted the importance of evaluating microbial counts alongside sensory attributes when assessing meat quality. These findings support the integrated quality assessment approach recommended by [41] and suggest that an APC limit of approximately 10^6^ CFU/g may serve as a more conservative and consumer-relevant indicator of product acceptability than the commonly cited 10^7^ CFU/g threshold [18].

Although microbial reductions were modest, encapsulated *V. sinaiticum* extract delayed microbial growth, reduced pH increases, and improved the retention of color and sensory attributes during storage. Therefore, its practical application is likely to be most effective as part of a multi-hurdle preservation strategy in combination with packaging technologies or other natural antimicrobial systems rather than as a standalone preservative.

Encapsulation systems (MD, GA, MDGA) and plant extract treatments provided a moderate delay in microbial growth and sensory degradation, likely due to controlled release and stabilization of phenolic bioactive compounds [44,48,49]. However, these treatments did not fully prevent spoilage during extended refrigerated storage.

Overall, the results demonstrate that treatment type significantly influenced microbial and sensory stability of beef. Encapsulated and extract-treated samples exhibited improved quality retention compared to the control; however, the effect was partial. Thus, microencapsulation of *V. sinaiticum* extract can be considered a supportive preservation strategy that delays, but does not completely inhibit spoilage under refrigerated conditions.

## 3. Materials and Methods

### 3.1. Materials, Chemicals, and Reagents

*V. sinaiticum* leaves were collected following our previous work [3,4]. Beef loin (livestock grade 1) was purchased 24 h postmortem from a local meat packing store in Chuncheon, Gangwon Province, in July 2024, and sliced to 1.5 cm thickness. MD (dextrose equivalent 16.5–19.5) and GA were obtained from Sigma Aldrich Merck (Seoul, Republic of Korea). All other chemicals and reagents, including methanol, ethanol, sodium carbonate, sodium nitrite, sodium hydroxide, Folin–Ciocalteu reagent, potassium persulfate, catechin, gallic acid, aluminum chloride, and sodium chloride, were of analytical grade and also purchased from Sigma Aldrich Merck, Seoul, Republic of Korea.

### 3.2. Encapsulated Extract: Preparation and Characterization

#### 3.2.1. Extraction of Plant Material

The maceration extraction (ME) of *V. sinaiticum* was adapted from [4]. Dried leaves were extracted with 70% ethanol (solvent-to-material ratio 1:30 m/V) at 30 °C for 24 h in a shaking water bath (JSSB-50 T, JS Research Inc., Gongju, Republic of Korea) based on preliminary analysis establishing its optimal polarity to extract a broad spectrum of bioactive phytochemicals. The supernatant was collected, wrapped in aluminum foil, and stored at −80 °C until further analysis.

#### 3.2.2. Total Polyphenol Content (TPC)

The TPC was determined using the Folin–Ciocalteu method as described by [3] with minor modifications. Briefly, 0.2 mL of the extract was mixed with 2.5 mL of 10% (*v*/*v*) Folin–Ciocalteu reagent, followed by the addition of 2 mL of 7.5% (*w*/*v*) sodium carbonate solution. The mixture was incubated at 50 °C for 10 min and subsequently cooled at room temperature. The absorbance was measured at 750 nm using a Spectra i3x multi-mode microplate reader (Molecular Devices, LLC, Seoul, Republic of Korea). TPC was quantified using a gallic acid standard curve and expressed as mg gallic acid equivalents per gram of dry weight (mg GAE/g dw).

#### 3.2.3. Preparation and Freeze-Drying of Encapsulated *V. sinaiticum* Extract

The encapsulation matrix was prepared using MD and GA at a ratio of 8:2 (*w*/*w*), selected based on preliminary optimization for encapsulation efficiency and stability [3,50]. A 10% (*w*/*w*) MD solution was prepared by dissolving MD (10 g) in distilled water (90 g) under continuous agitation (70 rpm) at 27 °C for 12 h. A 4% (*w*/*w*) GA solution (4 g GA in 96 g distilled water) was freshly prepared 2 h before use. The two solutions were combined and homogenized using a magnetic stirrer (Heidolph MR 3001 K, Heidolph Instruments GmbH & Co. KG, Schwabach, Germany) at 1250 rpm to obtain a final total solid content of 10%.

Dried *V. sinaiticum* leaves were extracted, and the resulting extract was centrifuged (4000 rpm, 15 min) to remove insoluble materials. The supernatant was concentrated using a rotary evaporator (40 °C, 100 mbar) and subsequently freeze-dried (−50 °C, 0.1 mbar, 72 h) to obtain a stable phenolic-rich powder. For encapsulation, the freeze-dried extract was dispersed in the coating solution at a coating-to-core ratio of 10:1 (*w*/*w*) and homogenized using an Ultra-Turrax T25 (IKA-Werke GmbH & Co. KG, Staufen, Germany) (12,000 rpm) to ensure uniform distribution (Table 5). The resulting emulsion was frozen at −80 °C for 2 h and freeze-dried (Model FDU-7003, Operon Co., Ltd., Gimpo-si, Republic of Korea) at −55.8 °C and 0.40 mbar for 72 h. The obtained powder was ground, passed through a 20-mesh sieve, and stored in light-protected, sealed aluminum containers at 4 °C until further analysis. All experiments were conducted in triplicate.

#### 3.2.4. Determination of Process Yield and Encapsulation Efficiency

The encapsulation process was conducted following the modified methodology of [50]. Briefly, the core *V. sinaiticum* extract and the respective wall material solutions (MD, GA, and MDGA) were homogenized to ensure a stable emulsion. The mixtures were then pre-frozen at −80 °C for 2 h (CLN-51U, Nihon Freezer, Tokyo, Japan) to facilitate rapid ice crystal formation. Lyophilization was performed using a laboratory-scale freeze-dryer (FDU-7003, Operon, Republic of Korea) operated at a condenser temperature of −55.8 °C and a vacuum pressure of 0.40 mbar for a duration of 72 h. This low-temperature sublimation process was specifically selected to preserve the thermolabile antimicrobial metabolites within the extract. The resulting porous cake was gently ground, sieved through a 20-mesh screen to ensure particle size uniformity, and stored in light-protected aluminum containers at 4 °C for further analysis.

Process yield (PY) was calculated according to [9] as follows:

(1) PY %=mpmdw of LFS×100,

where m_p_ is the recovered powder mass and mdw of LFS is the total dry matter of the liquid feed solution.

Encapsulation efficiency (EE) was determined by quantifying the TPC and surface phenolic content using standard spectrophotometric methods and expressed as the percentage of phenolic compounds successfully retained within the encapsulation matrix.

Encapsulation efficiency (EE) was calculated as:

(2) EE %=(WiWu)×100,

where W_i_ is the content of the target compound in microparticles, and W_u_ is its content in the initial feed solution.

Loading capacity (LC, mg/g dw) was determined as:

(3) LC %mg/g dw=(c×Vmi)×100,

where c is the concentration of the analyzed compound from the calibration curve, V is the volume of dissolved microparticles, and m_i_ is the dry mass of dissolved microparticles.

#### 3.2.5. Thermal Properties (DSC)

Thermal properties of MD, GA, their physical mixture (MDGA), and the encapsulated samples were analyzed using differential scanning calorimetry (DSC; Discovery DSC, TA Instruments, New Castle, DE, USA). Samples were heated from 20 to 300 °C at a rate of 10 °C/min under a nitrogen atmosphere (50 mL/min), following [51]. DSC analysis was conducted to evaluate the thermal stability of the encapsulated system, which is relevant to the retention of antimicrobial activity during thermal processing and storage conditions in meat preservation.

### 3.3. Meat Application Study

#### 3.3.1. Color Parameter: Beef

The color of beef samples containing encapsulated and extract forms of *V. sinaiticum* was evaluated according to the method of [3,19], with minor modifications. Color attributes of beef samples treated with *V. sinaiticum* crude leaf extract (free and encapsulated forms) were measured using a calibrated colorimeter (Minolta CR-400, Konica Minolta, Japan). Measurements were recorded in the CIE L*a*b* color space, where L* represents lightness, a* redness/greenness, and b* yellowness/blueness. The instrument was calibrated using a standard white reference plate prior to analysis, according to the manufacturer’s instructions. Before analysis, beef samples were equilibrated to room temperature. For each treatment group (control, free extract, and encapsulated formulations prepared with MD, GA, and their combination (MDGA)), color values were recorded at three different locations on the sample surface to account for heterogeneity. Each measurement was performed in triplicate, and results are expressed as mean ± standard deviation. For consistency, the color of dried *V. sinaiticum* leaf powder and the corresponding encapsulated powders was evaluated under the same conditions. The total color difference (ΔE) was calculated to assess perceptible differences among treatments.

#### 3.3.2. Texture Profile Analysis (TPA)

The TPA, including adhesiveness, hardness, chewiness, springiness, and cohesiveness, was analyzed using a texture analyzer (TA-X1, Lloyd Instruments Ltd., Fareham, UK). Samples measuring 1.5 cm in thickness and 2.5 cm in width were placed in the analyzer’s sample holder. The texture analyzer settings were as follows: pre-load speed of 10 mm/min, post-load speed of 2 mm/s, maximum cell load of 50 kg, and compression level of 30% [51].

#### 3.3.3. Antimicrobial Analysis—Disc Diffusion Assay

Antimicrobial activity was evaluated using the disc diffusion method with minor modifications to a previously reported protocol [23]. Test microorganisms included *Escherichia coli* O157:H7 (ATCC 43894), *Bacillus cereus* (ATCC 10987), *Staphylococcus aureus* (ATCC 13565), and *Candida albicans* (KCCM 11282). Each bacterial strain was cultured in Tryptic Soy Broth (TSB; Difco Laboratories Inc., Detroit, MI, USA) at 37 °C for 18 h. The inoculum was adjusted to approximately 10^6^–10^7^ CFU/mL (0.5 McFarland standard), and 100 µL was uniformly spread onto Mueller–Hinton agar plates (for bacteria). For *C. albicans*, an appropriate medium (Sabouraud Dextrose Agar) was used. Sterile paper discs (7 mm diameter) were impregnated with 30 µL of each test sample, including free and encapsulated *V. sinaiticum* leaf extracts, and placed onto the inoculated agar surface. Ciprofloxacin (20 µg/mL; 30 µL/disc) served as the positive control, while the negative control consisted of the corresponding solvent or encapsulation matrix without extract (same volume per disc: 0.5 mL, 1 mL, 1.5 mL, and 2 mL). Plates were incubated at 37 °C for 24 h (bacteria) and at appropriate conditions for *C. albicans*. Antimicrobial activity was expressed as the diameter of the inhibition zone (mm), measured including the disc diameter. All assays were performed in triplicate.

### 3.4. Evaluation of Free and Encapsulated V. sinaiticum Extract on Beef Quality During Storage at 4 °C

The effect of *V. sinaiticum* leaf extract and its encapsulated forms on beef quality was evaluated during refrigerated storage at 4 ± 2 °C for 15 days. Beef samples were individually treated by surface application of the extracts before storage. Briefly, samples were divided into five groups: (i) control (untreated), (ii) free extract, (iii) MD, (iv) GA, and (v) combined carrier system (MDGA). The treatment solutions were applied by uniform surface coating at a standardized concentration, ensuring complete coverage of the beef loin surface. After application, samples were allowed to stand for a defined contact time to ensure absorption and adherence of the treatment before packaging. All samples were then packed in low-density polyethylene (LDPE) film (0.01 mm thickness; oxygen transmission rate: 35,273 cc/m^2^/24 h) and stored under refrigerated conditions (4 ± 2 °C). Beef quality was monitored throughout storage by evaluating pH, microbiological load, and sensory attributes, including aroma and color, at pre-determined sampling intervals following established food safety guidelines [23,41,43]. It was hypothesized that encapsulated formulations would provide enhanced antimicrobial protection and improved preservation of sensory quality compared to free extract and untreated controls.

#### 3.4.1. pH

pH measurements were taken using an Orion 230A pH meter (Thermo Fisher Scientific, Waltham, MA, USA). A 10 g portion of the beef loin sample was homogenized with 90 mL of distilled water for 30 s before analysis.

#### 3.4.2. Microbiological Properties

A 10 g portion of beef loin was aseptically transferred into a sterile stomacher bag and homogenized with 90 mL of sterile 0.1% peptone water to obtain an initial 10^−1^ dilution. Homogenization was performed using a BagMixer 400 stomacher (Interscience, Saint-Nom-la-Bretèche, France) for 2 min. Serial decimal dilutions were prepared in sterile peptone water. Aerobic Plate Count (APC) and *Escherichia coli* enumeration were performed using 3M Petrifilm plates [52] (3M Microbiology, Saint Paul, MN, USA) according to the manufacturer’s instructions. Plates were incubated aerobically at 37 °C for 48 h. Colonies were counted on plates containing 30–300 CFU and expressed as log CFU/g. *E. coli* was identified based on typical blue colonies associated with gas production. All microbiological determinations were performed in duplicate/triplicate, and mean values were used for statistical analysis.

#### 3.4.3. Sensory Characteristics (Aroma and Color)

Sensory evaluation was conducted by a screened and trained panel of 15 members, comprising 7 males and 8 females aged 20–45 years. Panelists were trained following ISO 8586:2023 [53] guidelines to harmonize the evaluation of beef quality attributes, with specific emphasis on aroma and color acceptability. The evaluated samples included untreated control beef, beef treated with free *V. sinaiticum* extract, and beef treated with encapsulated formulations prepared using MD, GA, and MDGA.

Ethical approval for the involvement of human participants was obtained from the Institutional Review Board of Debre Berhan University (Protocol No. DBU/IRB/015/2024). In accordance with the Declaration of Helsinki, all participants were informed about the study objectives, the food-grade nature of the ingredients, and potential allergens. Written informed consent was obtained from each panelist before the commencement of the sensory evaluation.

Evaluations were performed at designated storage intervals under controlled environmental conditions (21 ± 1 °C and 60% relative humidity) in individual sensory booths. To minimize expectation bias, samples were coded with random three-digit numbers and presented to panelists in a randomized order. Aroma and color acceptability were assessed using a 9-point hedonic scale, where 9 indicated “extremely like” and 1 indicated “extremely dislike,” following standard sensory evaluation protocols for meat products.

### 3.5. Statistical Analysis

Statistical analysis was performed using JMP Pro version 17 (SAS Institute Inc., Cary, NC, USA). Data were analyzed using analysis of variance (ANOVA) to evaluate the effects of treatment and storage time. A two-way ANOVA model (treatment × storage time) was applied to assess main and interaction effects. When significant differences were detected (*p* < 0.05), means were separated using Tukey’s HSD post hoc test. Results are presented as mean ± standard deviation.

## 4. Conclusions

Encapsulation of *V. sinaiticum* leaf extract using GA, MD, and their combination (MDGA) improved the physicochemical stability of the extract and contributed to maintaining selected quality attributes of beef during storage. Thermal analysis indicated enhanced stability of the encapsulated systems, while treated samples showed better retention of color and acceptable textural properties compared to the control. Encapsulated extracts also demonstrated inhibitory effects against *Escherichia coli* and *Staphylococcus aureus*, with some variation among wall materials. Overall, the findings suggest that encapsulation may support the functional application of plant-derived extracts in meat preservation. However, the extent of antimicrobial effectiveness and shelf-life improvement appears to be formulation and condition-dependent, indicating the need for further validation under real storage and processing conditions.

## Figures and Tables

**Table 1:** Extraction yield, TPC, and Process yield, encapsulation efficiency, and loading capacity of *V. sinaiticum* extract.

*V. sinaiticum* Extract			
Extraction		Yield	TPC
70% ethanol		25.85 ± 0.08 ^a^	181.50 ± 0.12 ^a^
Encapsulation			
Encapsulant type	PY (%)	EE (%)	LC (mg GAE/g dw)
MD	42.5 ± 1.2 ^a^	78.3 ± 2.5 ^a^	18.5 ± 0.8 ^a^
GA	51.8 ± 1.5 ^b^	85.1 ± 1.9 ^b^	21.2 ± 0.7 ^a^
MDGA (8:2 *w*/*w*)	54.7 ± 1.3 ^b^	92.5 ± 1.7 ^b^	24.3 ± 0.9 ^a^

TPC: total phenolic content; PY: process yield; EE: encapsulation efficiency; LC: loading capacity; mg GAE/g DW: milligrams of gallic acid equivalents per gram of dry powder. Different superscript letters within the same column indicate statistically significant differences among treatments (*p* < 0.05).

**Table 2:** Texture profile analysis of beef samples coated with *V. sinaiticum* extract and its microencapsulated forms, MD, GA, and blend during refrigerated storage (0–9 days) compared with an untreated control.

Day	Texture	MD	GA	MDGA	Extract	Control at 4 °C Temperature
	Ad	0.444 ± 0.004 ^a^	0.575 ± 0.032 ^a^	0.3117 ± 0.24 ^a^	0.234 ± 0.005 ^a^	0.246 ± 0.006 ^b^
	Sp	0.0093 ± 0.001 ^a^	0.023 ± 0.023 ^ab^	0.02 ± 0.01 ^a^	0.011 ± 0.005 ^a^	0.017 ± 0.001 ^a^
1	Co	0.0067 ± 0.002 ^b^	0.006 ± 0.0006 ^b^	0.005 ± 0.001 ^b^	0.007 ± 0.002 ^c^	0.0063 ± 0.002 ^b^
	Gu	2.766 ± 0.014 ^b^	3.842 ± 0.010 ^b^	4.416 ± 0.125 ^b^	3.018 ± 0.010 ^b^	3.101 ± 0.163 ^b^
	Ch	2.663 ± 0.019 ^b^	3.823 ± 0.003 ^b^	4.336 ± 0.006 ^b^	3.635 ± 0.050 ^b^	3.644 ± 0.035 ^b^
	Ad	0.167 ± 0.007 ^b^	0.0957 ± 0.004 ^b^	0.1427 ± 0.003 ^a^	0.314 ± 0.006 ^a^	0.398 ± 0.013 ^a^
	Sp	0.007 ± 0.001 ^b^	0.008 ± 0.001 ^b^	0.008 ± 0.001 ^b^	0.008 ± 0.002 ^a^	0.009 ± 0.002 ^b^
3	Co	0.025 ± 0.009 ^a^	0.028 ± 0.004 ^a^	0.020 ± 0.010 ^b^	0.022 ± 0.002 ^b^	0.018 ± 0.003 ^a^
	Gu	11.729 ± 0.009 ^a^	8.647 ± 0.006 ^a^	24.660 ± 0.06 ^a^	34.832 ± 0.201 ^a^	25.478 ± 0.028 ^b^
	Ch	8.039 ± 3.580 ^a^	6.467 ± 1.758 ^a^	17.378 ± 8.69 ^a^	23.629 ± 13.373 ^a^	17.854 ± 4.269 ^a^
	Ad	0.334 ± 0.003 ^c^	0.325 ± 0.005 ^c^	0.0273 ± 0.007 ^a^	0.100 ± 0.005 ^a^	0.384 ± 0.005 ^a^
	Sp	0.0048 ± 0.004 ^c^	0.016 ± 0.012 ^b^	0.008 ± 0.001 ^b^	0.005 ± 0.002 ^a^	0.009 ± 0.001 ^b^
9	Co	0.011 ± 0.001 ^b^	0.029 ± 0.004 ^a^	0.172 ± 0.002 ^a^	0.055 ± 0.005 ^a^	0.018 ± 0.007 ^a^
	Gu	11.729 ± 0.02 ^a^	8.647 ± 0.05 ^a^	24.660 ± 0.05 ^a^	34.832 ± 0.06 ^a^	25.478 ± 0.07 ^a^
	Ch	8.039 ± 0.03 ^a^	6.467 ± 0.07 ^a^	17.378 ± 0.07 ^a^	23.629 ± 0.03 ^a^	17.854 ± 0.05 ^a^

MD: maltodextrin, GA: gum arabic and MDGA: a combination of maltodextrin–gum arabic blend. Ad: Adhesiveness, Sp: Springiness, Co: Cohesiveness, Gu: Gumminess, Ch: Chewiness. Different letters within the same column are significantly different (*p* < 0.05).

**Table 3:** Changes in color parameters (CIE L*, a*, b*, and ∆E) of beef samples treated with *V. sinaiticum* extract and its microencapsulated forms (MD, GA, and blend [MDGA]) during refrigerated storage (0–15 days) compared with an untreated [control].

Parameter		Storage (Days)					
Color	Treatment	1	3	6	9	12	15
CIE L*	Control	35.09 ± 0.99 ^a^	35.01 ± 0.01 ^a^	33.68 ± 1.01 ^a^	29.69 ± 0.99 ^b^	28.8 ± 0.95 ^b^	26.04 ± 1.01 ^c^
	MD	37.36 ± 0.99 ^a^	35.43 ± 0.99 ^ab^	35.46 ± 1.01 ^ab^	33.90 ± 0.05 ^bc^	32.44 ± 1.02 ^c^	28.89 ± 1.02 ^d^
	MDGA	37.53 ± 1.01 ^a^	35.87 ± 1.01 ^ab^	35.39 ± 0.99 ^ab^	34. 45 ± 1.01 ^bc^	32.3 ± 0.99 ^c^	28.72 ± 0.98 ^d^
	GA	37.62 ± 0.98 ^a^	35.96 ± 0.99 ^a^	35.24 ± 1.01 ^ab^	33.14 ± 0.99 ^bc^	31.19 ± 1.00 ^c^	28.22 ± 1.03 ^d^
	Extract	36.08 ± 1.00 ^a^	35.84 ± 1.01 ^a^	33.68 ± 0.99 ^a^	29.69 ± 1.01 ^b^	28.8 ± 0.95 ^b^	28.03 ± 0.99 ^b^
CIE a*	Control	21.3 ± 0.98 ^a^	18.55 ± 0.78 ^b^	10.32 ± 1.03 ^c^	9.06 ± 1.01 ^c^	8.93 ± 0.99 ^c^	8.8 ± 0.95 ^c^
	MD	18.07 ± 1.01 ^a^	15.43 ± 0.98 ^a^	10.15 ± 1.02 ^b^	9.56 ± 0.95 ^bc^	7.42 ± 0.98 ^c^	7.42 ± 1.01 ^c^
	MDGA	18.58 ± 0.34 ^a^	15.5 ± 0.1 ^b^	10.27 ± 0.21 ^c^	9.33 ± 0.1 ^d^	8.93 ± 0.02 ^d^	7.17 ± 0.02 ^e^
	GA	18.97 ± 0.9 ^a^	15.73 ± 0.98 ^b^	10.15 ± 0.99 ^c^	9.54 ± 0.99 ^cd^	9.17 ± 0.98 ^cd^	7.18 ± 0.96 ^d^
	Extract	19.44 ± 0.91 ^a^	18.20 ± 0.89 ^a^	10.17 ± 0.92 ^b^	9.03 ± 1.01 ^b^	8.95 ± 1.00 ^b^	8.45 ± 0.91 ^b^
CIE b*	Control	7.66 ± 1.01 ^a^	6.58 ± 1.0 ^ab^	6.40 ± 1.01 ^ab^	6.17 ± 1.01 ^ab^	5.57 ± 1.02 ^ab^	4.07 ± 1.0 ^b^
	MD	8.15 ± 1.02 ^a^	7.93 ± 0.98 ^ab^	7.47 ± 1.01 ^ab^	7.36 ± 0.99 ^ab^	6.10 ± 0.83 ^ab^	5.51 ± 0.98 ^b^
	MDGA	7.39 ± 0.98 ^a^	6.58 ± 0.99 ^a^	6.53 ± 0.93 ^a^	6.04 ± 0.99 ^a^	5.94 ± 1.58 ^a^	5.68 ± 0.96 ^a^
	GA	8.17 ± 0.99 ^a^	7.92 ± 0.98 ^a^	7.68 ± 1.01 ^a^	7.45 ± 1.01 ^a^	7.41 ± 1.00 ^a^	7.39 ± 1.03 ^a^
	Extract	6.60 ± 0.90 ^a^	6.45 ± 0.98 ^a^	6.33 ± 1.06 ^ca^	6.14 ± 1.04 ^a^	5.35 ± 0.98 ^a^	3.93 ± 1.00 ^a^
∆E	Control	63.04 ± 0.94 ^a^	60.19 ± 1.04 ^b^	50.36 ± 0.97 ^c^	44.85 ± 0.98 ^d^	43.15 ± 0.98 ^d^	38.85 ± 0.97 ^e^
	MD	61.5 ± 1.15 ^a^	59.80 ± 0.91 ^a^	53.05 ± 0.98 ^b^	50.89 ± 1.02 ^b^	45.93 ± 1.0 ^c^	41.17 ± 0.99 ^d^
	MDGA	61.53 ± 0.6 ^a^	56.08 ± 1.08 ^b^	50.49 ± 1.01 ^c^	47.54 ± 0.97 ^d^	42.35 ± 0.98 ^e^	39.02 ± 0.99 ^f^
	GA	62.46 ± 0.66 ^a^	59.71 ± 0.95 ^b^	53.06 ± 0.76 ^c^	50.16 ± 0.96 ^d^	47.80 ± 1.10 ^d^	42.95 ± 0.98 ^e^
	Extract	62.76 ± 0.97 ^a^	59.65 ± 1.28 ^b^	50.3 ± 0.95 ^c^	44.7 ± 0.9 ^d^	42.80 ± 1.05 ^d^	38.60 ± 0.95 ^e^

*V. sinaiticum*: *Verbascum sinaiticum*, CIE L*: Lightness, CIE a*: Red-Green, CIE b*: Yellow-Blue, ∆E: difference in color: untreated: Control, MD: maltodextrin, GA: gum arabic, and MDGA: blended. Different letters within the same column are significantly different.

**Table 4:** Changes in pH, microbiological counts, and sensory attributes (aroma) of treated beef loin samples during refrigerated storage at 4 °C.

Parameter	Storage Day					
	1	3	6	9	12	15
pH						
Control	5.64 ± 0.01 ^d^	5.70 ± 0.02 ^d^	5.85 ± 0.03 ^c^	6.05 ± 0.04 ^c^	6.28 ± 0.06 ^b^	6.62 ± 0.08 ^a^
MD	5.63 ± 0.02 ^e^	5.66 ± 0.01 ^e^	5.74 ± 0.02 ^d^	5.90 ± 0.03 ^c^	6.10 ± 0.05 ^b^	6.34 ± 0.07 ^a^
MDGA	5.62 ± 0.01 ^e^	5.64 ± 0.01 ^e^	5.70 ± 0.01 ^d^	5.85 ± 0.02 ^c^	6.02 ± 0.03 ^b^	6.20 ± 0.05 ^a^
GA	5.63 ± 0.01 ^e^	5.67 ± 0.02 ^e^	5.76 ± 0.02 ^d^	5.92 ± 0.03 ^c^	6.12 ± 0.04 ^b^	6.30 ± 0.06 ^a^
Extract	5.64 ± 0.01 ^d^	5.68 ± 0.02 ^d^	5.80 ± 0.02 ^c^	5.96 ± 0.04 ^c^	6.20 ± 0.05 ^b^	6.45 ± 0.07 ^a^
Aerobic plate count (Log CFU/g)						
Control	3.04 ± 0.17 ^e^	3.60 ± 0.06 ^d^	3.79 ± 0.02 ^d^	5.94 ± 0.18 ^c^	7.01 ± 0.19 ^b^	8.26 ± 0.03 ^a^
MD	2.94 ± 0.15 ^e^	3.40 ± 0.04 ^d^	3.60 ± 0.08 ^d^	5.80 ± 0.06 ^c^	6.89 ± 0.19 ^b^	7.89 ± 0.04 ^a^
MDGA	2.97 ± 0.16 ^e^	3.49 ± 0.03 ^d^	3.65 ± 0.02 ^d^	5.85 ± 0.02 ^c^	6.95 ± 0.25 ^b^	7.96 ± 0.07 ^a^
GA	3.01 ± 0.18 ^e^	3.50 ± 0.05 ^d^	3.71 ± 0.03 ^d^	5.90 ± 0.09 ^c^	6.99 ± 0.19 ^b^	7.95 ± 0.06 ^a^
Extract	3.02 ± 0.19 ^e^	3.52 ± 0.03 ^d^	3.71 ± 0.05 ^d^	5.92 ± 0.09 ^c^	7.00 ± 0.35 ^b^	8.01 ± 0.05 ^a^
*E. coli* content (×10^5^ CFU/g, mean ± SD)						
Control	5.48 ± 0.17 ^d^	5.90 ± 0.12 ^cd^	6.02 ± 0.18 ^bcd^	6.31 ± 0.19 ^bc^	6.57 ± 0.21 ^ab^	6.93 ± 0.30 ^a^
MD	5.22 ± 0.13 ^b^	5.29 ± 0.09 ^b^	5.45 ± 0.12 ^b^	5.84 ± 0.17 ^a^	5.91 ± 0.21 ^a^	6.23 ± 0.15 ^a^
MDGA	5.32 ± 0.12 ^c^	5.59 ± 0.06 ^c^	5.60 ± 0.07 ^c^	6.12 ± 0.13 ^b^	6.22 ± 0.13 ^b^	6.60 ± 0.15 ^a^
GA	5.43 ± 0.06 ^d^	5.69 ± 0.05 ^c^	5.77 ± 0.05 ^c^	6.27 ± 0.07 ^b^	6.37 ± 0.07 ^b^	6.75 ± 0.13 ^a^
Extract	5.29 ± 0.09 ^c^	5.57 ± 0.07 ^bc^	5.84 ± 0.144 ^b^	5.92 ± 0.09 ^b^	6.53 ± 0.16 ^a^	6.86 ± 0.26 ^a^
Aroma						
Control	8.50 ± 0.18 ^a^	8.00 ± 0.30 ^a^	6.70 ± 0.02 ^b^	5.80 ± 0.32 ^c^	2.70 ± 0.17 ^d^	1.60 ± 0.29 ^e^
MD	8.50 ± 0.20 ^a^	8.20 ± 0.27 ^a^	6.75 ± 0.01 ^b^	5.90 ± 0.21 ^c^	2.95 ± 0.14 ^d^	1.75 ± 0.32 ^e^
MDGA	8.50 ± 0.18 ^a^	8.10 ± 0.14 ^b^	6.85 ± 0.04 ^c^	5.80 ± 0.32 ^d^	2.90 ± 0.14 ^e^	1.68 ± 0.20 ^f^
GA	8.50 ± 0.18 ^a^	8.25 ± 0.17 ^a^	7.0 ± 0.01 ^b^	5.85 ± 0.32 ^c^	2.85 ± 0.13 ^d^	1.70 ± 0.14 ^e^
Extract	8.50 ± 0.18 ^a^	7.85 ± 0.21 ^a^	6.80 ± 0.04 ^b^	5.85 ± 0.24 ^c^	2.80 ± 0.14 ^d^	1.65 ± 0.17 ^e^

Control: *V. sinaticum* extract, MD: Maltodextrin, GA: gum arabica and MDGA: blended. Different letters (a–e) within the same row letters assigned to mean values indicate statistically significant differences (*p* < 0.05) within parameters over storage days.

**Table 5:** Formulation of the microencapsulation system.

Coating Material	Ratio MD:GA (*w*/*w*)	DW (Distilled Water)	Ratio Coating Material: Core Mixture Ratio (*w*/*w*)
100% MD	(10:0)	90	10:1
100% GA	(0:4)	96	10:1
100% (MD + GA)	(8:2)	90	10:1

MD: maltodextrin, GA: gum arabica and blend (MDGA), DW: distilled water.

## Data Availability

Data are available upon request from the corresponding author.
